# Characterizing plasma albumin concentration changes in TB/HIV patients on anti retroviral and anti –tuberculosis therapy

**DOI:** 10.1186/s40203-014-0003-9

**Published:** 2014-09-16

**Authors:** Kuteesa R Bisaso, Joel S Owen, Francis W Ojara, Proscovia M Namuwenge, Apollo Mugisha, Lawrence Mbuagbaw, Livingstone S Luboobi, Jackson K Mukonzo

**Affiliations:** 1 Department of Pharmacology & Therapeutics, College of Health Sciences, Makerere University, Kampala, Uganda; 2 School of pharmacy, Union University Tennessee, Tennessee, USA; 3 Centre for Operational Research in Africa, Kampala, Uganda; 4 Department of Clinical Chemistry, Mulago National Referral Hospital, Kampala, Uganda; 5 Department of Clinical Epidemiology and Biostatistics, McMaster University, Ontario, Canada; 6 Department of Mathematics, College of Natural Sciences, Makerere University, Kampala, Uganda; 7 CIHR Canadian HIV Trials Network, Vancouver, BC Canada

**Keywords:** NONMEM, Disease progression modeling, Semi-mechanistic model, Mathematical model, Albumin, Tuberculosis, HIV, Anti Retroviral therapy, Efavirenz, Rifampicin

## Abstract

**Purpose:**

Plasma albumin, a biomarker for hepatic function, is reported to correspondingly decrease in concentration as disease severity increases in chronic infections including HIV and TB. Our objective was to develop a semi-mechanistic disease progression model to quantify plasma albumin concentration changes during TB and HIV therapy and identify the associated covariate factors.

**Methods:**

Plasma albumin concentration data was collected at specified times for 3 months from 262 HIV participants receiving efavirenz based anti retroviral therapy. Of these, 158 were TB co-infected and on Rifampicin based anti –tuberculosis co-treatment. An indirect response model with zero order albumin production and first order elimination was developed in NONMEM version 7.2 to describe our data. Genotype (CYP2B6*6 and 11, CYP3A5, ABCB1c.3435C>T and ABCB1rs), TB disease status, baseline age, body weight, plasma creatinine, alanine transaminase enzyme and CD4^+^ count were the potential model covariates tested.

**Results:**

The proposed model successfully described plasma albumin concentration changes in the study population. There was a 10.9% and 48.6% increase in albumin production rates in HIV only and TB co-infected participants respectively. Participants co-infected with TB showed a 44.2% lower baseline albumin secretion rate than those without TB while ABCB1c.3435C>T mutation was associated with a 16% higher steady state albumin secretion rate following treatment.

**Conclusion:**

A semi-mechanistic model describes plasma albumin concentration changes in HIV patients on ART. Further work is required to establish the utility of the model in monitoring disease progression and predicting prognosis in HIV and TB co-infected patients in absence of or during treatment.

**Electronic supplementary material:**

The online version of this article (doi:10.1186/s40203-014-0003-9) contains supplementary material, which is available to authorized users.

## 1 Background

In humans, albumin is the most abundant plasma protein (55-60% of plasma protein) having a normal plasma concentration of 3.5-5 g/dl (Bircher et al. [1991]). It is exclusively synthesized in the liver. In normal adults, albumin is synthesized and released at a zero-order rate of 157–230 mg per kilogram body weight per day into an exchangeable pool of 3.5-5 grams per kilogram body weight. Approximately 38–45% of albumin is intravascular (Nicholson et al. [2000]). Albumin has a half life of 15–20 days (Nicholson et al. [2000], Beeken et al. [1962]) and elimination mainly occurs in the muscles, skin and kidney (60-80%) and the liver and intestinal tract (10% each) (Tavill [1972]).

Among other functions, albumin binds ligands and transports endogenous and exogenous substances, including several drugs in blood (Tavill [1972]). Increase in plasma albumin is associated with significant reduction in intracellular penetration and effectiveness of highly bound antiretroviral drugs like efavirenz (Avery et al. [2013], Boffito et al. [2003]).

Reduced plasma albumin concentrations have been reported in states of chronic infection such as TB, HIV, Hep B and C (Olawumi and Olatunji [2006], Akinpelu et al. [2012], Zia and Shankar [2012]) and as a result, there are suggestions for its use as a prognostic marker in pretreated HIV and TB patients (Feldman et al. [2003], Graham et al. [2007], Sudfeld et al. [2013], Alvarez-Uria et al. [2013]). Increased catabolism of albumin due to inflammation, worsening of nutritional status as well as a direct degenerative effect on the liver are reported as the probable causes of reduction of plasma albumin in states of chronic infection (Bircher et al. [1991], Kaysen et al. [2002]). Owing to simplicity in terms quantification and low cost of plasma albumin determination, it could substitute CD4+ and viral load tests (Graham et al. [2007], Kannangai et al. [2008]) as a prognostic marker during care for HIV and co morbidities such as TB.

A number of modeling techniques have shown robustness and have been applied in prediction of drug concentrations, pharmacodynamic outcomes and disease progression using biomarkers. We developed and validated a semi-mechanistic non-linear mixed effects model describing changes in plasma albumin concentration in HIV and TB patients. The model was used to study changes in albumin production by the liver and the associated covariate factors.

## 2 Methods

### 2.1 Data description

The current clinical study was nested in a PhD project (Mukonzo [2011]) and utilized secondary data. The data consisted of 262 ART naïve HIV patients, 158 of whom were co-infected with tuberculosis. The patients were started on combination ART comprised of Efavirenz, lamivudine and zidovudine. Those with TB co-infection had been started on anti-TB treatment (2 months ethambutol/isoniazid/rifampicin/pyrizinamide, followed by 4 months of isoniazid and rifampicin) at least 2 weeks prior to starting ART. Blood samples were collected on days 1, 3, 7, 14, 21, 42, 56, and 84 after ART initiation and serum albumin concentrations measured using Abbott Aeroset Bromocresol Green (BCG) (Abbot, Maidenhead, Berkshire, UK) method. In addition, baseline body weight, age, CYP3A5, CYP2B6*6, CYP2B6*11, ABCB1c.3435C>T and ABCB1 c.4046A>G genotype were determined. The study procedure was approved by The Uganda National Council for Science and Technology. Informed consent was obtained from the participants and the study was carried out according to the provisions of the Declaration of Helsinki. Details on study participants and data collection were previously reported (Mukonzo [2011]). Waiver of consent was obtained from Institutional review board of the School of Biomedical Sciences, Makerere University College of Health Sciences to use the data. Also, the data were analyzed anonymously.

### 2.2 Model development

**Figure 1:**
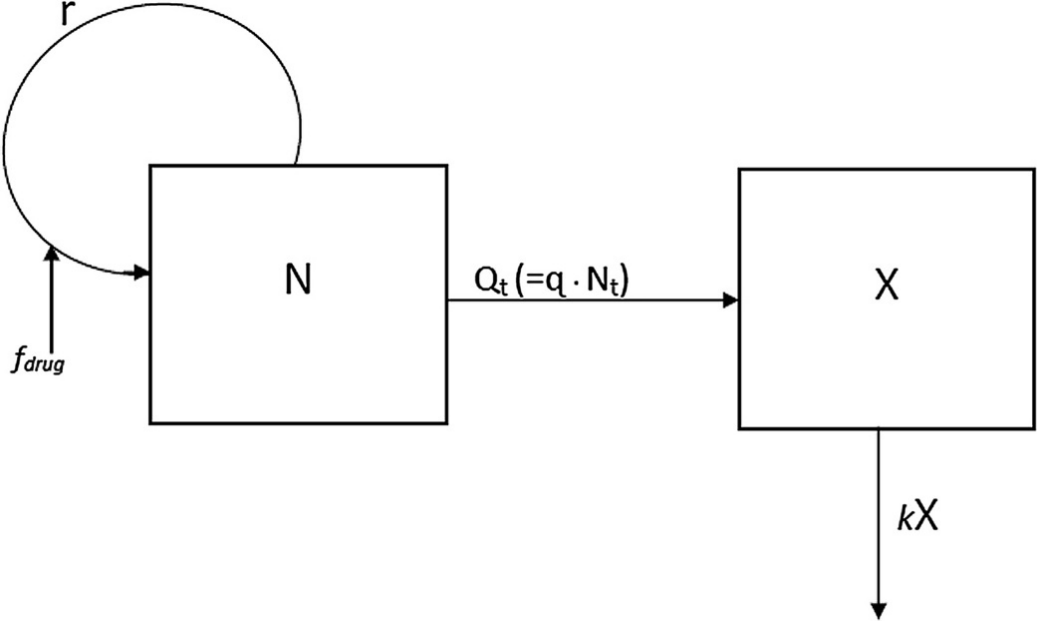
**Structure of the disease progression model describing plasma albumin concentration changes in TB-HIV patients.** r is the rate of change of hepatocyte population, *N* (*N*_*t*_) is the Hepatocyte population at time t, *X* is the plasma albumin concentration at time t, *k* is the albumin elimination rate constant, *f*_*drug*_ is the effect of ART on the change in hepatocyte population, *Q*_*t*_ is the total albumin secretion rate at time t, is the albumin secretion rate per hepatocyte.

The model consists of the hepatocyte compartment (N) which secretes albumin at a zero order rate (*Q*) (g/dl/hr) into the central plasma compartment (*X*) from which elimination occurs by first order process with an elimination rate constant *K* (1/hr). Figure 1. The production compartment assumes a constant average albumin production rate (q) per hepatocyte such that a change in hepatocyte population results in proportional changes in the albumin production and secretion rate. Thus, if *N*_*(t)*_ is the hepatocyte population at a time *t* (hr), the albumin production rate at that time is given by:

1 $$Q_{\left(t\right)}=q\cdot N_{\left(t\right)}$$

The hepatocyte population is assumed to change according to the Verhulst’s self limiting logistic equation (Murray [2002]).

2 $$\frac{\mathrm{dN}}{\mathrm{dt}}=\mathrm{rN}\left(1-\frac{N}{N_{\mathrm{ss}}}\right)$$

At t=0, N= *N*_0_.

As t→∞, N=*N*_*ss*_

*N*_*ss*_ is the size of the stable steady state population as determined by the overall health and nutritional status following initiation of anti-TB treatment and HAART. *N*_*ss*_ may be bigger or smaller than *N*_*0*_*.* The rate at which *N*_*ss*_ is reached is measured by *r* (1/hr). ART and anti-TB treatment alter the natural progression of HIV and TB diseases respectively thus modifying the degenerative process of the liver. ART and anti TB treatment have a protective disease dependent effect on the liver. If *f*_*drug*_ is the function describing the combined efficacy of treatment, *f*_*drug*_ reflects exposure in terms of plasma drug concentrations or doses. It causes a disease dependent modification of the degenerative effect. According to Post (Post [2009]), the rate of change of Q during treatment is given by:

3 $$\frac{\mathrm{dQ}}{\mathrm{dt}}=\mathrm{rQ}\left(1-\frac{Q}{Q_{\mathrm{ss}}}\right)\cdot f_{\mathrm{drug}}$$

Due to absence of pharmacokinetic data in these patients, the rate of change of hepatocyte population (r) and drug effect (*f*_*drug*_) were combined into one parameter R = r ∙ *f*_*drug*_*.*Therefore changes in albumin secretion rate are described by the equation;

4 $$\frac{\mathrm{dQ}}{\mathrm{dt}}=\mathrm{RQ}\left(1-\frac{Q}{Q_{\mathrm{ss}}}\right)$$

Thus, if *Q*_*0*_ is the albumin secretion rate at the start of treatment and *Q*_*ss*_ is the steady state albumin secretion rate following treatment with a specified regimen, albumin secretion rate at any time *t* during treatment with the same regimen is given by:

5 $$Q_{\left(t\right)}=\frac{Q_{\mathrm{ss}}Q_{0}e^{\mathrm{Rt}}}{\left[Q_{\mathrm{ss}}+Q_{0}\left(e^{\mathrm{Rt}}-1\right)\right]}\to Q_{\mathrm{ss}}\;\mathrm{as}\;t\to \infty$$

Due to sparseness of the data (on average 3 observations per individual), the kinetics of albumin were modeled using a simplified one compartment with first order elimination model to minimize the number of parameters to be estimated. The change in elimination rate was assumed to be negligible and therefore not modeled.

6 $$\frac{\mathrm{dX}}{\mathrm{dt}}=Q_{\left(t\right)}-\mathrm{kX}$$

If X_0_ is the plasma albumin concentration at the start of treatment, solving *Equation 6* and substituting for Q_(t)_, the plasma albumin concentration at time ***t*** is given by:

7 $$X_{\left(t\right)}=X_{0}e^{-\mathrm{kt}}+\frac{Q_{0}Q_{\mathrm{ss}}e^{\mathrm{Rt}}\left(1-e^{-\mathrm{kt}}\right)}{k\left[Q_{\mathrm{ss}}+Q_{0}\left(e^{\mathrm{Rt}}-1\right)\right]}$$

At the start of treatment, X is given by X_0_ = Q_0_/k hence, the important parameters to be estimated are *Q*_*0*_*, Q*_*ss*_*, R* and *k.*

### 2.3 Data analysis

The model was fitted to the data and parameters estimated in a nonlinear mixed effects (“population”) analysis using NONMEM software version 7.2 (Beal and Sheiner [1980], Boeckmann et al. [1994]). The albumin elimination rate constant K was fixed to the literature value of 0.0336/day corresponding to a half life of 20.6 days. The population model parameter estimates were the fixed effects. Inter-individual variability in the parameters was modeled as log-normal distribution. The residual error was modeled as proportional but additive and additive plus proportional error models were also tested. The First Order Conditional Estimation (FOCE) method was used for the estimation.

### 2.4 Covariate analysis

Stepwise covariate analysis was performed using an automated method implemented in PSN software (Lindbom et al. [2004]). The effects of baseline body weight, TB disease status, CYP3A5, CYP2B6*6, CYP2B6*11, ABCB1c.3435C>T and ABCB1c.4046A>G genotypes on parameters Q_0_, Q_SS_ and R were analyzed. CD4 count and viral load were analyzed as a time varying covariate on parameters Q_SS_ and R. Each covariate-parameter relationship was first tested in a univariate manner. Covariates with one degree of freedom were included in the forward selection (α =0.05) if they reduced the OFV by at least 3.84, corresponding to a p-value of <0.05, for a *χ*^2^ distribution. The full covariate model was reached when the addition of further covariate-parameter relationships did not decrease the OFV to the specified criteria. The covariate-parameter relationships were re-examined in the backward deletion step in a manner similar to the forward inclusion step but reversed and with stricter criteria, corresponding to a significance level of α = 0.01 (ΔOFV=6.63 for one less parameter). In addition, the improvement of the fit in the covariate model was also evaluated from the change in the inter-individual variability, residual variability and basic goodness-of-fit plots (weighted residuals versus predicted concentrations and time, Population predictions versus observed concentrations and time). The final model was used to estimate the parameters.

### 2.5 Model evaluation

The dataset was randomly split into two. The larger dataset of 174 individuals (approximately two thirds of the whole dataset) was used for model building and bootstrap validation. A non-parametric bootstrap analysis was performed as an internal model evaluation technique. One thousand bootstrap data sets were created from the model development data set and the developed model was fit to each bootstrap data set. The percentage of runs that minimized successfully and estimated the covariance matrix, together with summary statistics (n, mean, median, and standard deviation, minimum, maximum) for the distribution of each model parameter were obtained to determine bias in any parameter. The final model parameter estimates were compared to the median and the percentile 95% confidence intervals (CI) of the non-parametric bootstrap replicates of the final model.

A visual predictive check (VPC) was performed using the final covariate model to evaluate correspondence between prediction corrected measurements and the model. The distribution (median, 5^th^ and 95^th^ percentiles) of the observed albumin concentrations was calculated. The final covariate model and its parameter estimates were used to simulate 1000 new datasets and used to calculate 95% Confidence Intervals for the above mentioned median and percentiles. The median and the percentiles of the measured data were plotted together with the confidence intervals from the model. The VPC was stratified on TB disease status and ABCB1c.3435C>T genotype.

The final model was applied to the validation data set by fixing the final parameter estimates of the model obtained above as the initial parameter values for the validation model and setting MAXEVAL=0 in the $ESTIMATION step, so as to generate predicted concentrations at each time point using the validation dataset of 88 individuals.

The root mean prediction error (imprecision) and the mean percentage prediction error (bias) were obtained according to the method proposed by Sheiner and Beal ([1981]).

## 3 Results

Out of the 262 participants 99 had only baseline (t=0) data. The average number of observations per individual participants was three (3). The demographic characteristics of the study population are summarized in Table 1.

**Table 1:** **Demographic characteristics of the study populations**

	HIV patients receiving HAART (n=262)		
	ALL	HIV + TB (n=158)	HIV only (n=104)
Female (% age)	52.9	49.5 (n=74)	61.5 (n =64)
Weight/kg	51 (47 – 58)	50.0 (45.0 – 53.0)	55.0 (50.0 – 60.0)
Age/years	33 (29 – 39)	31 (28 – 37)	37 (31 – 42)
CD4 cell count/ml	97 (40 – 179)	57 (21 – 137)	147 (89 – 207)
CD4 cells/ml at 12 weeks	216 (112 – 291)	194 (93 – 277)	247 (167 – 319)
ALT/Ul^−1^	18.0 (12 – 28.5)	23.9 (13.6 – 32.6)	14.0 (11 – 21)
ALB/gdl^−1^	3.02 (2.35 – 3.85)	2.57 (2.13 – 2.97)	3.91 (3.38 – 4.31)
ABCB1 3435CC	205	119	86
ABCB1 3435CT	56	38	18
ABCB1 3435TT	1	1	0
CYP2B6*6 (*1/*1)	116	81	35
CYP2B6*6 (*1/*6)	119	64	55
CYP2B6*6 (*6/*6)	27	13	14
CYP3A5 (*0/*0)	59	33	26
CYP3A5 (*0/*1)	130	86	44
CYP3A5 (*1/*1)	73	39	34

The genetics data is presented as number of participants with a given genotype. The other characteristics are presented as baseline median values with the inter-quartile range in brackets.

The random effects on parameters Q_ss_ and R had high shrinkage (>40%) and had a very low variability of less than 10^−6^ and were therefore dropped from the model.

**Figure 2:**
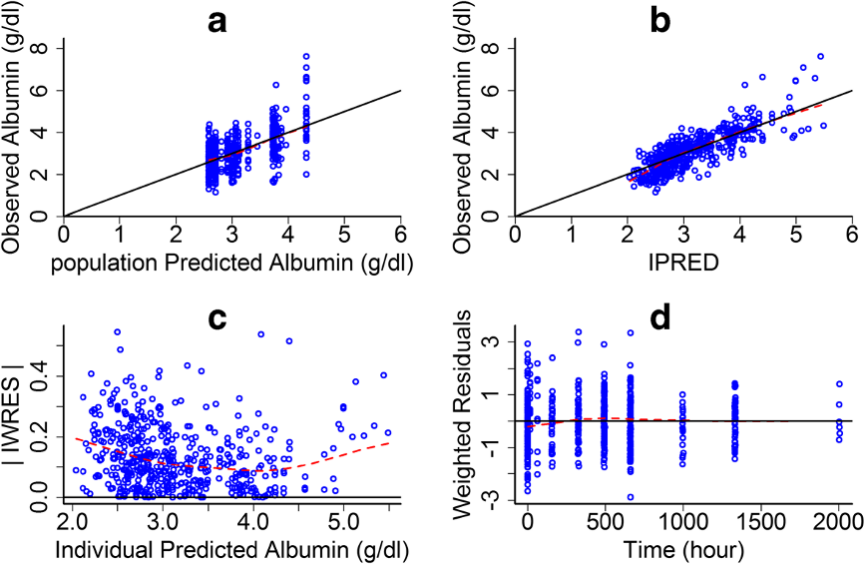
**Basic goodness of fit plots from the final covariate model.** Measured albumin concentrations are plotted against the population-fitted albumin concentration **(a)** and the individually fitted albumin concentration **(b)**. The solid line represents the line of identity and the red broken lines, smooth locally weighted least-squares regression. Absolute individual weighted residuals are plotted against the individually fitted albumin concentration **(c)** and the conditional weighted residuals are plotted against time **(d)**. Broken lines, a locally weighted least-squares regression; solid lines, lines of identity.

The final model described the dataset well as shown by plots of observation versus predictions (Figure 2a, 2b). The plot of individual weighted residuals versus individual prediction (Figure 2c) and the plot of weighted residuals versus time (Figure 2d) show a horizontal scatter indicating that the residual error distribution is adequately handled (Karlsson and Savic [2007]).

The parameter estimates of the base model are presented in Table 2. There is a 33% increase in albumin secretion rate among these patients when ART is initiated. Additional file 1: Table S1 shows base model parameters estimated separately for TB co-infected and HIV only patients. Following treatment, albumin secretion rate was predicted to increase to Q_ss_ which is 9% lower in TB-HIV patients than in those with HIV only indicating improvement in hepatocyte function in both groups. The estimated rate of increase in hepatocyte function(R) is 25% higher in TB co-infected patients as compared to those with HIV only. However, these differences between the two groups were not statistically significant. Covariate analysis was performed using an automated procedure with Perl speaks NONMEM (PsN) software. The final model retained TB disease status and ABCB1c.3435C>T genotype as a significant covariates on Q_0_. TB patients have a 44.2% less Q_0_ than those without TB while patients with one or more mutations in the ABCB1c.3435C>T gene have a 16% higher Q_0_ than those with homozygous wild type (ABCB1 3435CC). The final model improved the fit relative to the base model (ΔOFV = 101.57, d.f. = 2, p < 0.01). The final model parameter estimates are shown in Table 3.

**Table 2:** **Population disease progression parameter estimates for albumin dynamics in TB-HIV patients (base model)**

Parameter	HIV only		Description
	Mean	RSE (%)	
Q_0_ (g/dl/day)	0.1008	3	Baseline albumin secretion rate
Q_ss_ (g/dl/day)	0.1344	10	Steady state albumin secretion rate
R (1/day)	0.0096	34	Rate of change from Q_0_ to Q_ss_
K (1/day)	0.0336	FIX	Elimination rate constant for albumin
IIV_Q_0_ (%CV)	25.1	8	Inter-individual variability in baseline albumin secretion rate
Residual error (proportional) (%CV)	18.4	5	Variability in the residual error

**Table 3:** **Parameter estimates of full covariate model**

Parameter	Original dataset		Bootstrap datasets		
	Mean	*RSE (%)	Median	95% CI lower limit	95% CI upper limit
Q_0_ (g/dl/day)	0.0864	2	0.0864	0.084	0.0912
Q_0_ (HIV only)	0.1248	14	0.1248	0.108	0.1296
Q_0_ (ABCB1 mutation)	0.1008	34	0.1008	0.0864	0.1056
Q_ss_ (g/dl/day)	0.1464	16	0.1440	0.1176	0.3288
R (1/day)	0.0072	45	0.0072	0.001927	0.0144
K (1/day)	0.0336	FIX	0.0336	NA	NA
**Random effects parameters for both HIV only and TB-HIV**					
IIV_Q_0_ (%CV)	15.0	14	14.6	11.1	19.3
Residual error (proportional) (%CV)	18.2	5	18.1	16.8	19.8

*(NONMEM covariance step output).

Stability of the model was determined by use of non-parametric bootstrap technique using PsN. Of the 1000 bootstrap replicates, 950 minimized successfully were used to generate medians of parameters and percentile 95% confidence interval. As shown in Table 3, the mean parameter estimates obtained by fitting the final model to the data were similar to the median of the 950 bootstrap replicates and were contained within the 95% confidence interval, suggesting a high accuracy of NONMEM parameter estimates. The NONMEM parameter estimates also had moderate precision with relative standard errors of less than 50% for mean parameters and the random effects.

**Figure 3:**
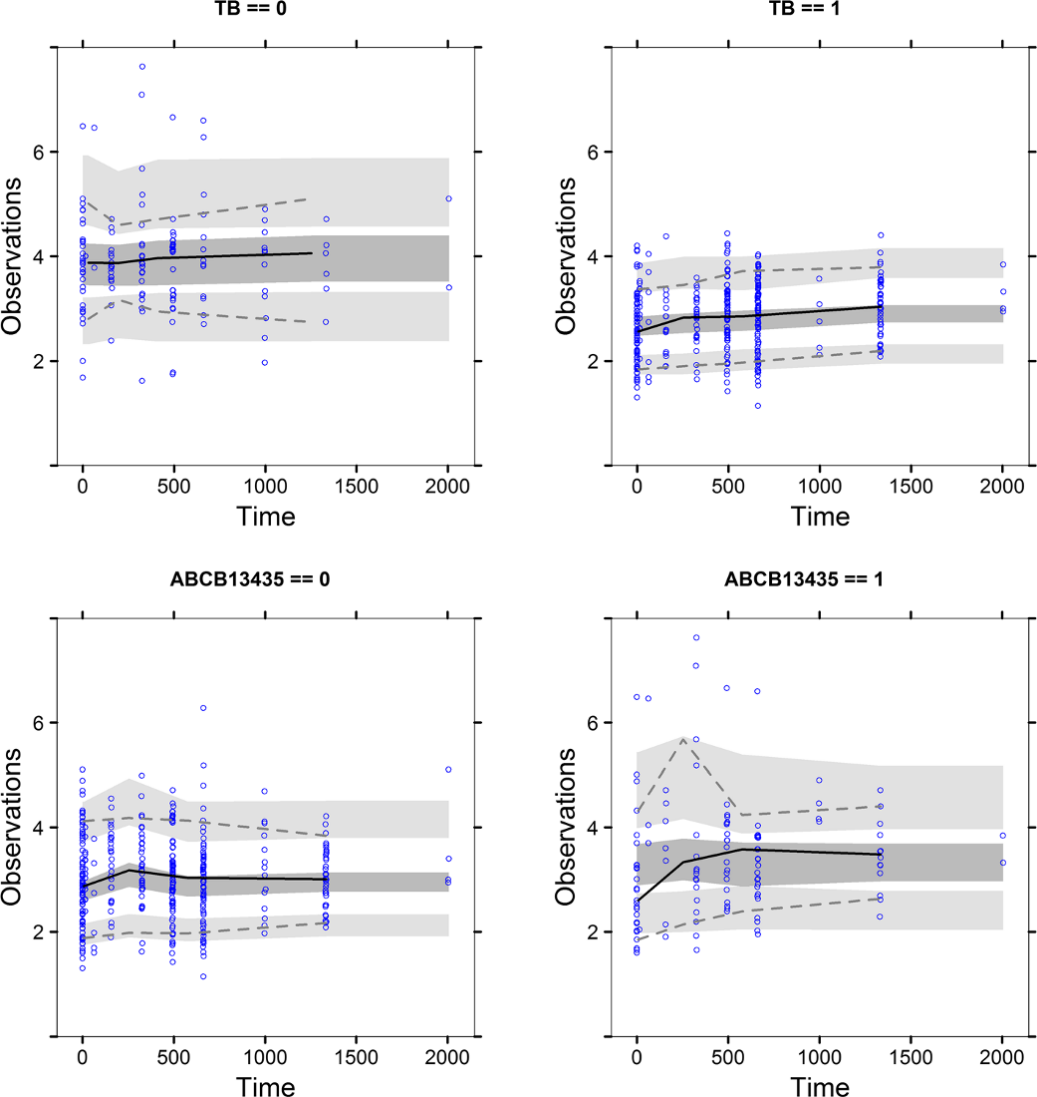
**A visual predictive check describing albumin kinetics, stratified on TB disease status (upper panel) and on ABCB1c.3435C>T genotype (lower panel).** Open circles, observed data points; broken lines, 5^th^ and 95^th^ percentiles; solid line, 50^th^ percentile; shaded areas, 95% confidence interval of the simulated (n=1000) 5^th^, 50^th^ and 95^th^ percentiles. ABCB13435==0 stands for ABCB1c.3435CC while ABCB13435==1 stands for ABCB1c.3435CT and ABCB1c.3435TT.

The validation dataset had 88 individuals and 269 observations. The mean prediction error was 0.012 with a 95% confidence interval of −0.063 to 0.087. The percentage root mean squared error which is a measure of how far the prediction error is from zero was 20.61%. Figure 3 is the visual predictive check of the final model. It shows a resemblance in trend and correspondence between observed and simulated data. The correspondence is stronger in patients with TB and HIV than in those with only HIV.

## 4 Discussion

Plasma albumin concentration is a function of its rate of synthesis, distribution and degradation. Hypoalbuminemia is a more common occurrence than hyperalbuminemia. Rapid changes in plasma albumin (occurring within hours) are most likely due to changes in elimination rate (fractional catabolic rate) or distribution of albumin as a result of either increased plasma water content or net movement into the interstitial space. However, because of its long half life, a sustained fall in albumin suggests clinically significant deterioration in its rate of synthesis by the liver (Kaysen et al. [2002], Bircher et al. [1991]). The present study utilized albumin concentration data collected over three months, therefore the model predictions are representative of chronic changes in albumin concentration.

The present model adequately describes the observed changes in albumin concentration and predicts population observation with minimum bias and error. The mean baseline albumin concentrations calculated from the estimated model parameters are similar to those observed. The baseline albumin secretion rate was significantly lower in patients co-infected with TB and HIV than in those with HIV only.

Individuals with ABCB1c.3435CC genotype had a 16% lower value of Q_0_ than those with ABCB1c.3435CT and ABCB1c.3435TT implying that presence of a mutation is associated with higher albumin secretion rates before treatment with ART. It is not immediately clear why this is the case since this single nucleotide polymorphism (SNP) has also been associated with predisposition to ART and rifampicin based anti-TB Drug Induced Liver Injury (DILI) through a possible low transport activity (Yimer et al. [2011], Thiebaut et al. [1987]).

Notably however is that when modeled as time varying covariates, neither CD4 count nor viral load had significant effects (p<0.05) on the model quantitative measures of disease progression and prognosis (R or Q_ss_). This is possibly explained by the fact that albumin concentrations improve secondary to overall health improvement upon initiation of HAART and anti-TB treatment. This therefore implies that although albumin may be a cheap and suitable prognostic marker for monitoring HIV disease, co-morbidities and ART, there is need for validation studies.

Disease progression modeling, a technique that was employed by the current study remains one of the most robust ways for prediction of associations and multiple covariate analysis. The model developed in this study is robust and has stable parameter estimates, satisfactorily describes the data and has a high predictive capacity. Nevertheless the model has limitation including the estimation of albumin kinetics using a simple one compartment model rather than the known two compartment kinetics as well as inability to model and estimate the variability. This was because of very sparse data which could not allow estimation of several parameters. We were also unable to model the change in albumin elimination rate partly because of the sparse data but also because our objective was to study the albumin production dynamics. Other limitations included the assumed negligible maturation time of new hepatocytes, as well as lag time between synthesis and secretion of albumin by hepatocytes as compared to the study period of three months.

Notwithstanding the limitations highlighted here, this model had high precision and low bias in prediction thus it can be used to predict plasma albumin concentration in individual patients. It is useful in predicting prognosis (Mehta et al. [2006]) and could be useful in describing pharmacokinetics of albumin bound drugs in these patients. In addition, our model provides a basis for extended models describing treatment effects, comparing different treatment regimens as well as accounting for direct drug toxicity on the liver.

## 5 Conclusion

In conclusion, the proposed one compartment semi-mechanistic model described changes in plasma albumin concentration following initiation of HAART in HIV patients with or without TB. Changes in albumin synthesis and secretion could influence changes in plasma albumin concentrations in patients on HAART. ABCB1c.3435C>T genotype and TB disease status are significantly associated with albumin secretion rates before initiation of ART in patients receiving HIV and TB co-treatment. The model could be useful in studying the variation in pharmacokinetic profiles of drugs that are highly protein bound in these patients during different stages of treatment with HAART. More work needs to be done establish the utility of this model in monitoring disease progression and predicting prognosis in HIV and TB patients.

## Additional file

## Electronic supplementary material

Additional file 1: Table S1.: Population disease progression parameter estimates for albumin dynamics in HIV and TB-HIV patients (base model). (PDF 421 KB)

Below are the links to the authors’ original submitted files for images.

Authors’ original file for figure 1

Authors’ original file for figure 2

Authors’ original file for figure 3

Authors’ original file for figure 4

## Abbreviations

ART: Anti retroviral therapy
HAART: Highly active Antiretroviral therapy
HIV: Human immunodeficiency virus
VPC: Visual predictive check
SNP: Single nucleotide polymorphism
TB: Tuberculosis
