# Erratum to: Characterizing plasma albumin concentration changes in TB/HIV patients on anti retroviral and anti –tuberculosis therapy

**DOI:** 10.1186/s40203-014-0005-7

**Published:** 2015-02-11

**Authors:** Kuteesa R Bisaso, Joel S Owen, Francis W Ojara, Proscovia M Namuwenge, Apollo Mugisha, Lawrence Mbuagbaw, Livingstone S Luboobi, Jackson K Mukonzo

**Affiliations:** Department of Pharmacology & Therapeutics, College of Health Sciences, Makerere University, Kampala, Uganda; School of pharmacy, Union University Tennessee, Tennessee, USA; Centre for Operational Research in Africa, Kampala, Uganda; Department of Clinical Chemistry, Mulago National Referral Hospital, Kampala, Uganda; Department of Clinical Epidemiology and Biostatistics, McMaster University, Ontario, Canada; Department of Mathematics, College of Natural Sciences, Makerere University, Kampala, Uganda; CIHR Canadian HIV Trials Network, Vancouver, BC Canada

## Erratum

As a result of continuous review of the model published in (Bisaso et al. 2014) corrections, clarifications and comparisons have been made.

1. We would like to retract the relation between ABCB1c.3435C > T gene mutation and baseline albumin secretion rate. This is because, even though the mutation in the gene was identified as a significant covariate with our data, we are still unable to biologically explain the relationship.
2. The final model equation (equation 7), referred to here as the “Simplified Solution” is not the direct solution of the differential equations preceding it. We would therefore like replace it with the unsolved differential equation below and show a comparison of the results of the two equations.

The unsolved differential equation has the initial value of X being Q_0_/k

$$ \frac{dX}{dt}=\frac{Q_0{Q}_{SS}{e}^{Rt}}{Q_{SS}+{Q}_0\left({e}^{Rt}-1\right)}-kX $$

The equation was fit to the data in NONMEM version 7.2, using a differential equations solver specified by the ADVAN6 and TOL = 3 subroutines. The First Order Conditional Estimation with interaction (FOCEI) estimation method was used.

The Interaction term was not used in the previous analysis because it has been reported not to be useful when there are small number of observations per individual and therefore does not provide different results (Peter 2011).

The two models, i.e. the previously defined simplified solution and the unsolved differential equation above were compared with respect to fit, difference and precision of parameter estimates, goodness of fit, prediction bias and precision as well as length of runtimes given the same initial parameter estimates.

As shown in Table 1 below, there was no significant difference in the fit of the models as measured by Objective function value (OFV < 3.84). The range of absolute difference in parameter estimates was 0.28% to 18% of the parameter estimates of the unsolved differential equation model above. The two models had similar relative standard errors of the estimates. The prediction bias and precision were also similar for both models. Both models were able to identify TB disease status and ABCB1c.3435C > T genotype as significant covariates on baseline albumin secretion rate. The goodness of fit plots and visual predictive check plots for both models were indistinguishable. As shown in Figure 1 and 2 below, the individual and population predictions of models were similar.

**Table 1:** **NONMEM estimated relative standard errors**, **percentage difference in parameter estimates**, **numerical predictive checks and other model comparison criteria for model1 and model2**

	**The unsolved differential equation model**	**The previous simplified solution**	**% parameter difference**
**Relative standard errors**			
Q_0_	2.6	2.5	0.28
Q_SS_	18.5	17.3	-0.16
R	48.2	46.0	17.78
Q_0__TB = 1(proportional increase in Q_0_ with TB)	13.9	14.1	1.27
IIV_Q_0_	18.0	17.8	0.73
Residual error	5.4	5.4	-0.31
**Other model criteria**			
OFV	57.151	58.457	NA
Condition Number	25.38	21.39	NA
runtime (seconds)	5.48	1.79	NA
**Measures of model prediction**			
Bias (%PE)	-0.66478	-0.66329	NA
Precision( 1-RMSE)	0.72481	0.72464	NA

**Figure 1:**
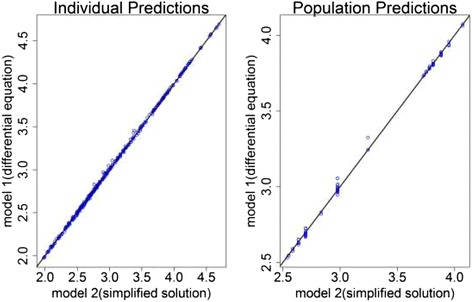
**Individual and population model predictions using model 1** **(differential equation)** **versus using model 2** **(simplified analytical solution.** The solid black line is the line of identity.

**Figure 2:**
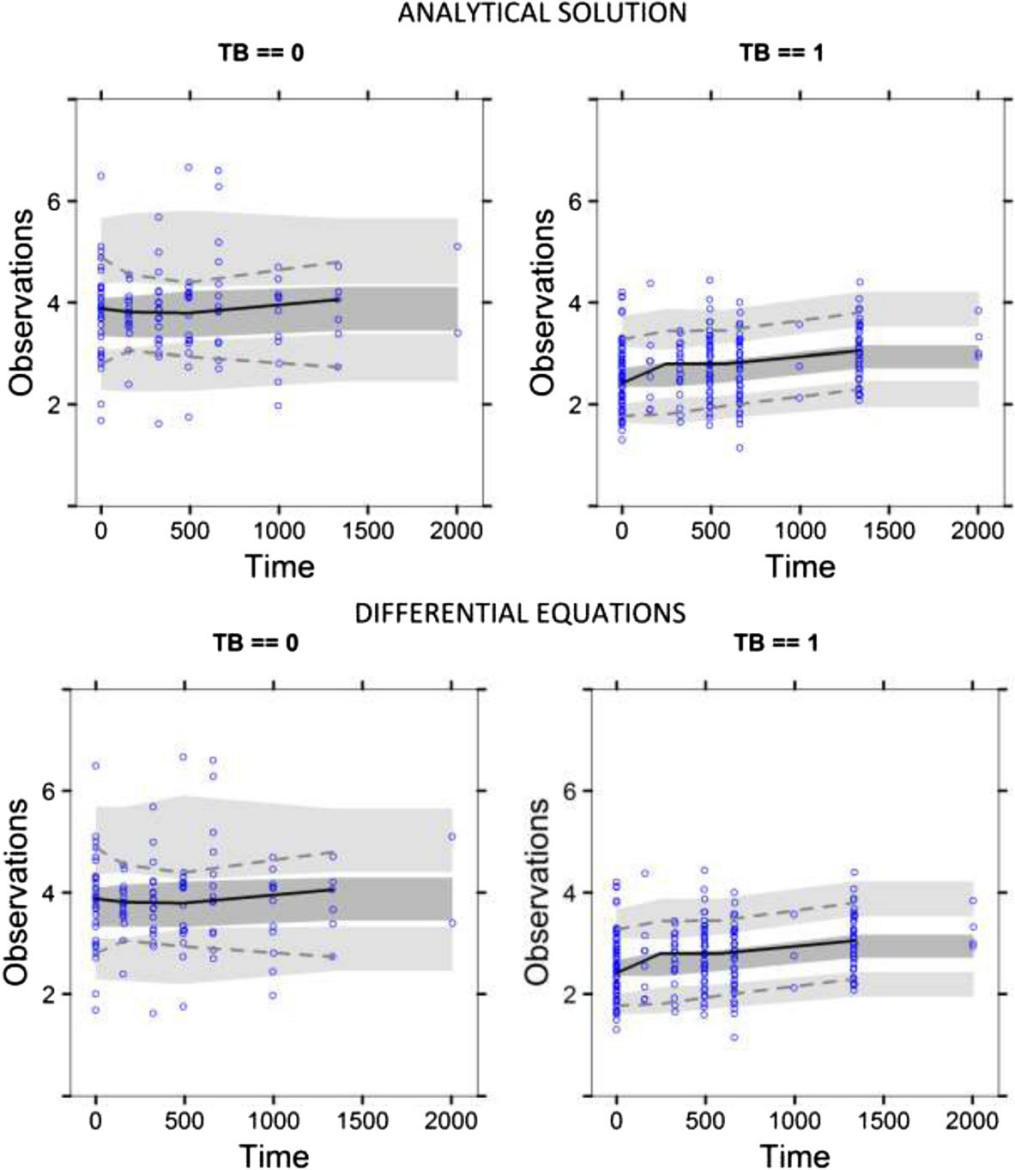
**Visual predictive checks of the two models,** **stratified on TB disease status.** Top panel has the VPC of the previous model and the bottom panel, the VPC of the unsolved equation.

The total run time (parameter estimation plus covariance) for the unsolved differential equations model above was more than three times that of the previously defined model and this ratio was higher during more intensive procedures like bootstrapping and stepwise covariate analysis.

## Conclusion

We would like to change model equation to the unsolved differential equation model. The model provided here and the previously defined model differ mostly in one parameter, R (rate of change of albumin secretion) and the computation times, but the other outputs including parameters, predictions and goodness of fit plots were similar.
