# Supplementary material for: Characterizing plasma albumin concentration changes in TB/HIV patients on anti retroviral and anti –tuberculosis therapy
Source: In Silico Pharmacol. 2014 Sep 16;2:3. doi: 10.1186/s40203-014-0003-9 (PMC4173069; doi:10.1186/s40203-014-0003-9)

Table 4. Population disease progression parameter estimates for albumin dynamics in TB and TB-HIV patients (base model).

parameter	HIV only		TB-HIV		Description
	**mean**	**RSE (%)**	**mean**	**RSE (%)**	
Q_0 (g/dl/hr)	0.0055	5	0.0037	2	Baseline albumin secretion rate
Q_{ss} (g/dl/hr)	0.0061	17	0.0055	17	Maximum albumin secretion rate
R (1/hr)	0.0003	69	0.0004	61	Rate of change from Q_0 to Q_{ss}
K (1/hr)	0.0014	FIX	0.0014	FIX	Elimination rate constant for albumin
**Random effects parameters for both HIV only and TB-HIV**					
IIV_Q_0	0.0269	12			Inter-individual variability in baseline albumin secretion rate
Residual error(proportional)	0.0335	5			Variability in the residual error

Table 5. Correlation matrix of the parameter estimates from the NONMEM covariance step.

	Q_0	Q_{ss}	R	OM11	RESIDUAL
**Q_0**	1.13E-4				
**Q_{ss}**	-2.72E-1	5.66E-4			
**R**	1.26E-1	-6.89E-1	1.45E-4		
**IIV_Q_0**	2.25E-1	-4.71E-1	5.26E-1	1.03E-3	
**RESIDUAL**	7.21E-2	-4.54E-2	-1.05E-2	2.7E-1	3.77E-3

Figure 4. VPC of the validation dataset.

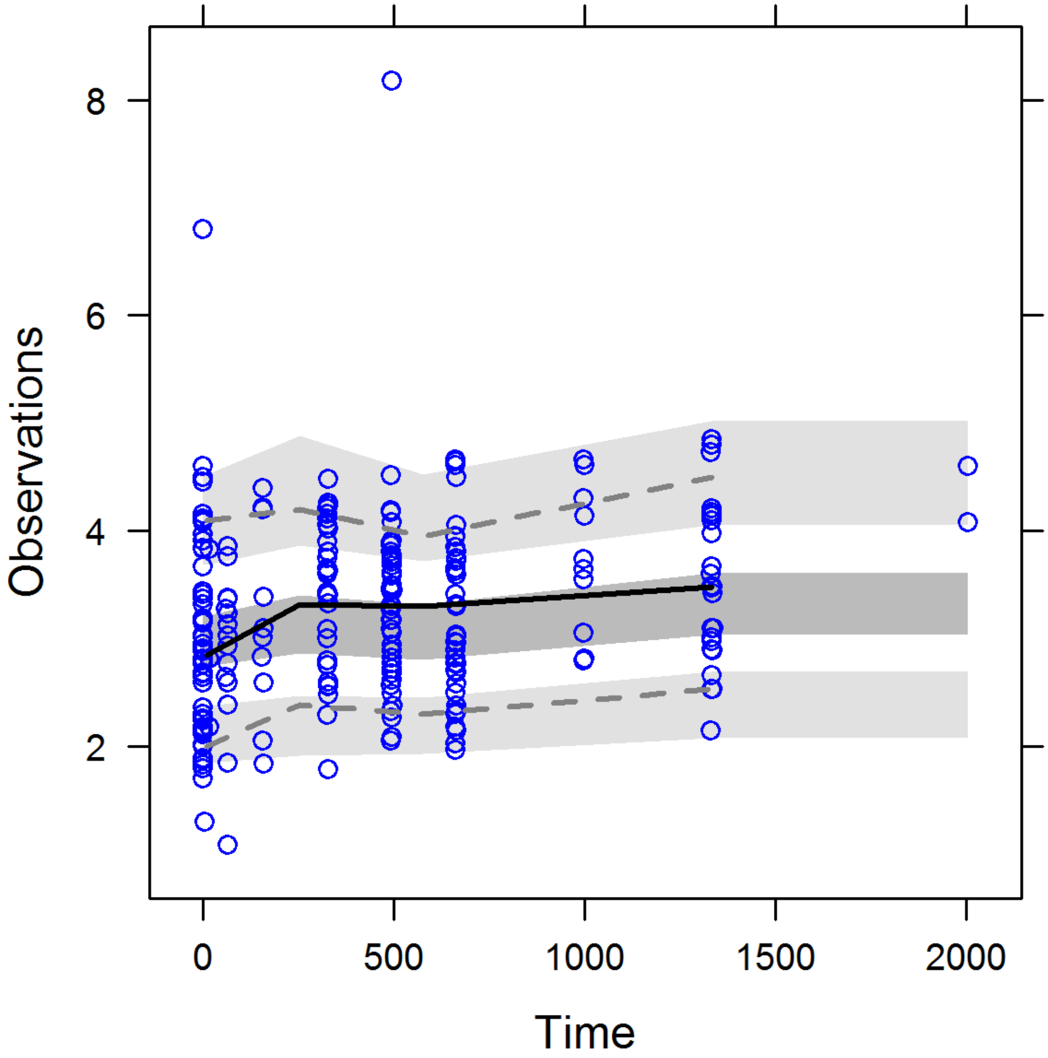

Figure 5. Parameter versus Parameter plots showing correlation between parameter estimates from 950 bootstrap estimates.

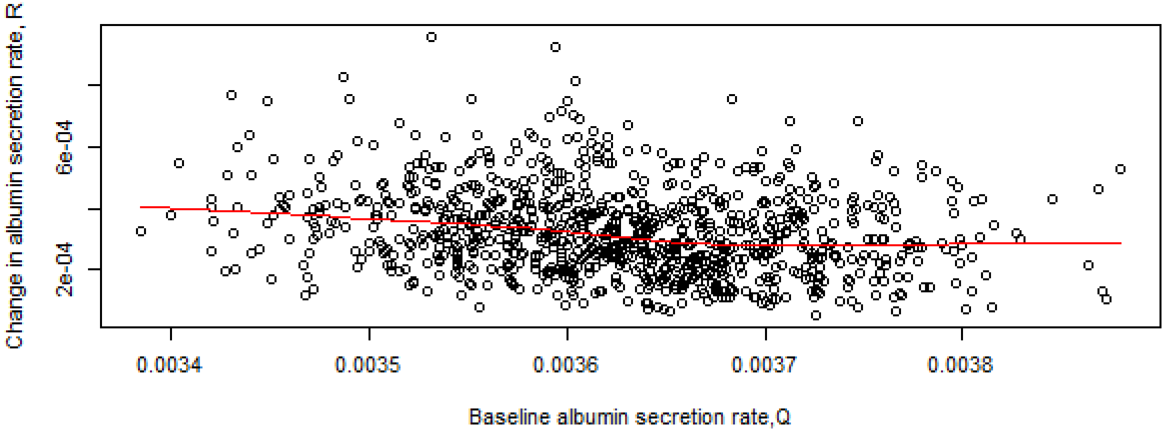

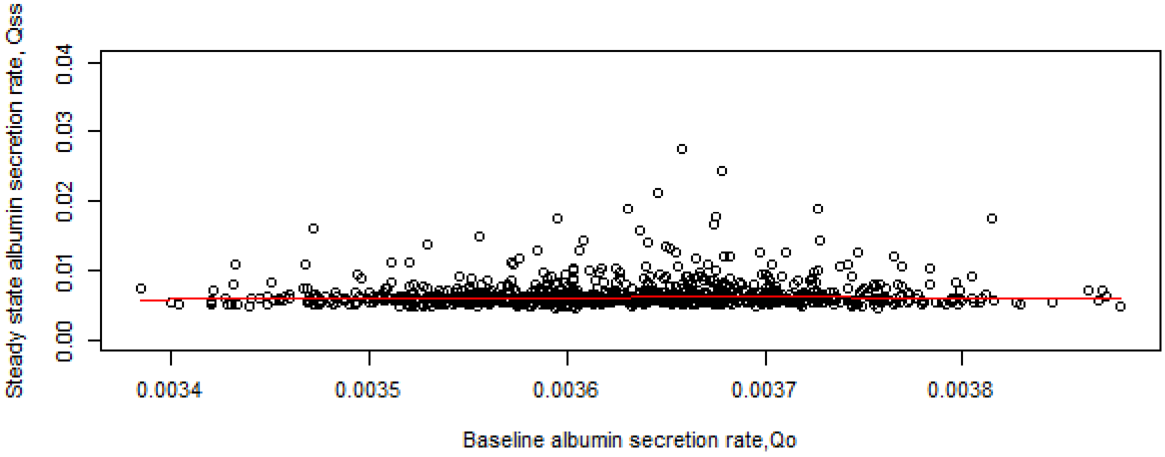

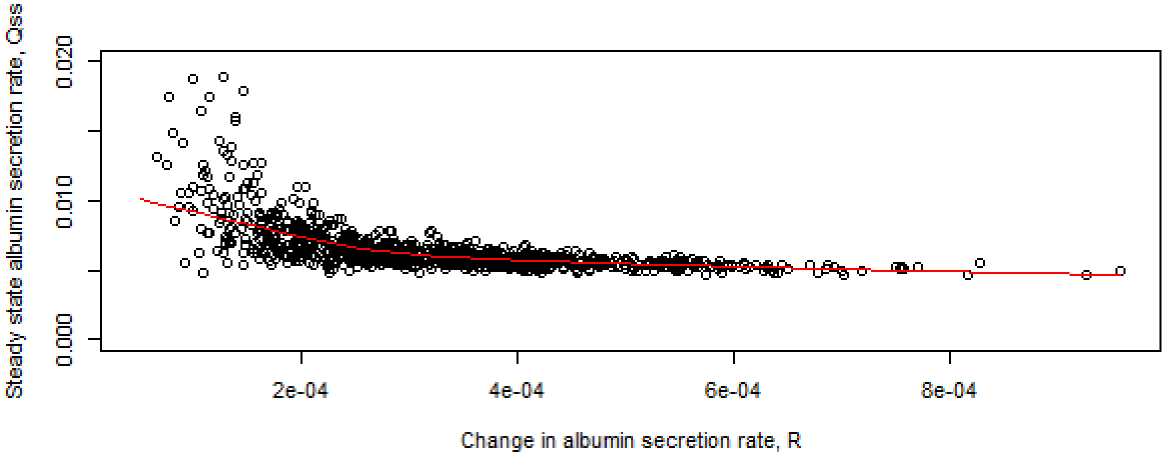

Figure 6. Inclusion frequence of covariates following 50 bootstraps of the stepwise covariate modeling.

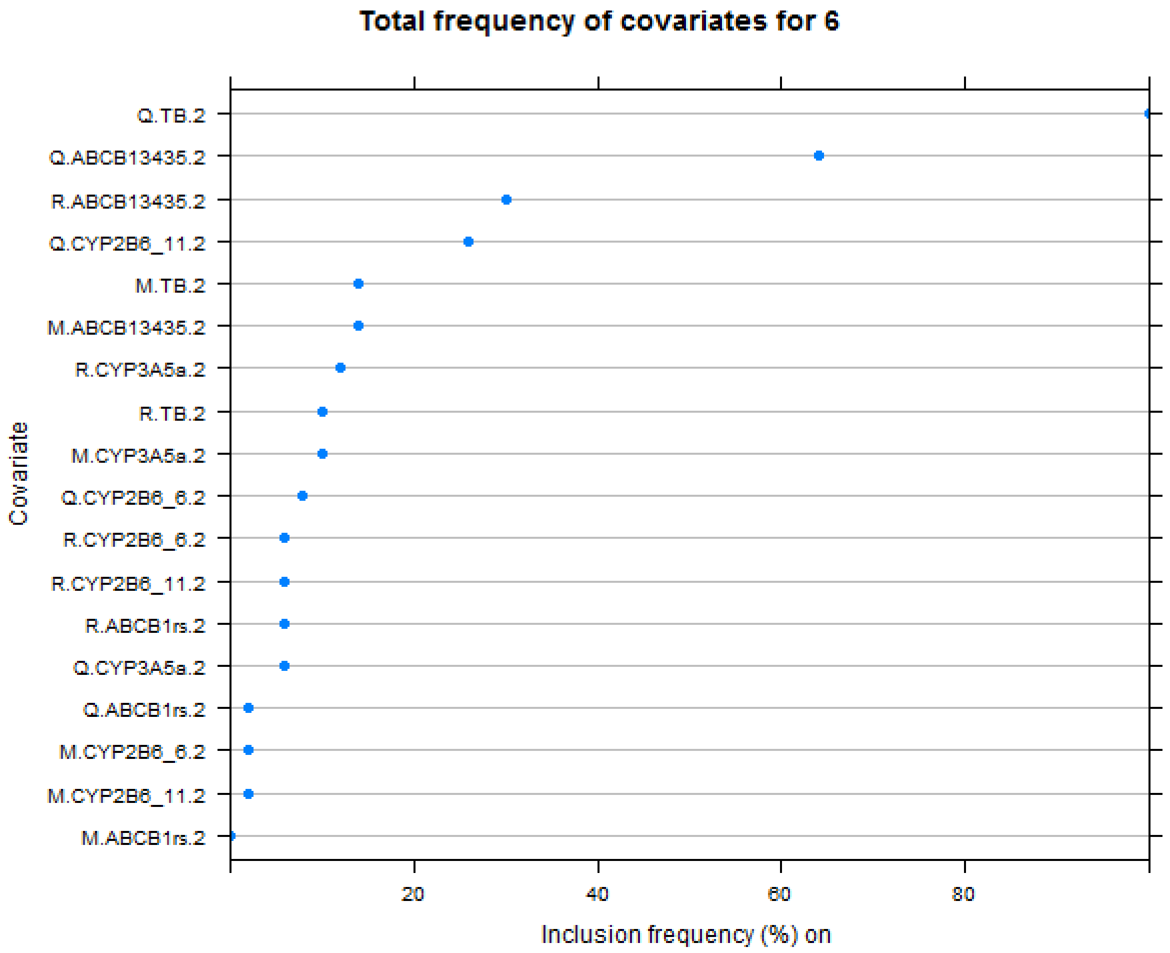

The Y axis represents " Parameter.covariate.relationship". e.g. Q.TB.2 means TB is a covariate on parameter Q with a proportional relationship. Q stands for Q_0 M stands Q_{ss}.

Figure 7. Goodness of fit plot using a simple exponential albumin production model.

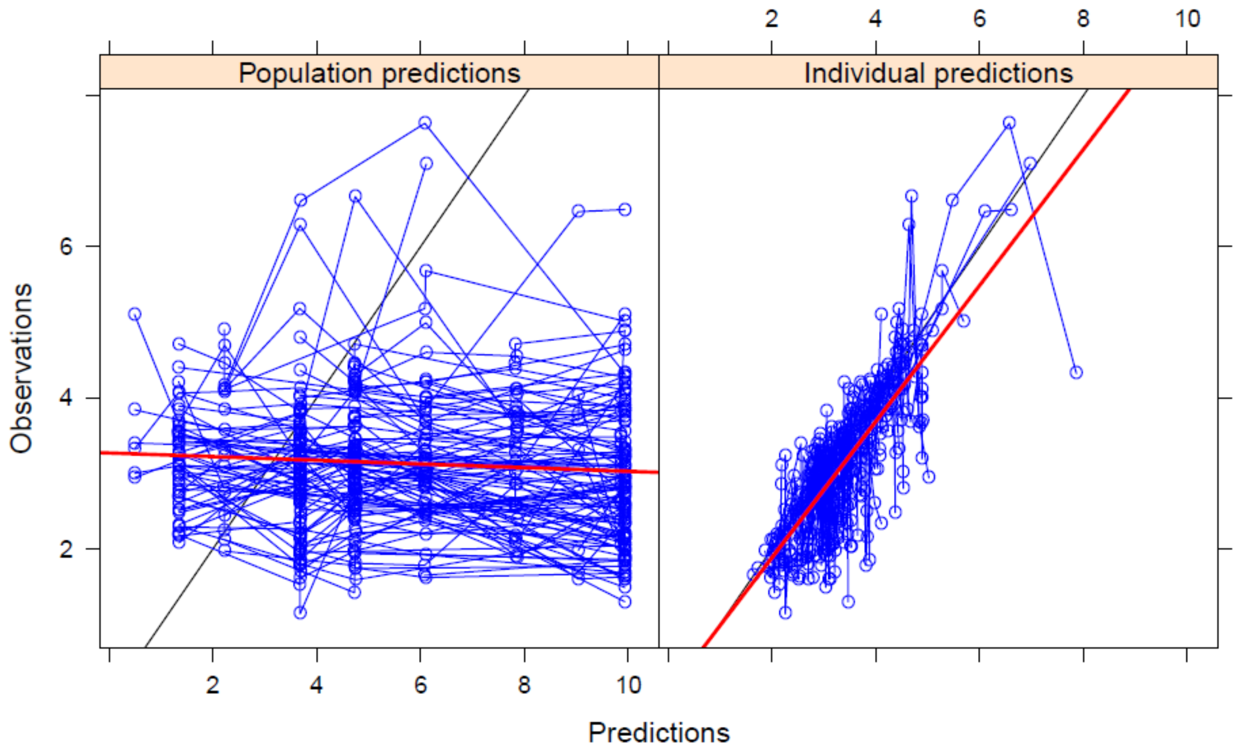

Supplement: Supplementary file 1 — Additional file 1: Table S1.: Population disease progression parameter estimates for albumin dynamics in HIV and TB-HIV patients (base model). (PDF 421 KB) [file 40203_2014_3_MOESM1_ESM.pdf]
