# Supplementary figures and images for: Characterizing plasma albumin concentration changes in TB/HIV patients on anti retroviral and anti –tuberculosis therapy
Source: In Silico Pharmacol. 2014 Sep 16;2:3. doi: 10.1186/s40203-014-0003-9 (PMC4173069; doi:10.1186/s40203-014-0003-9)

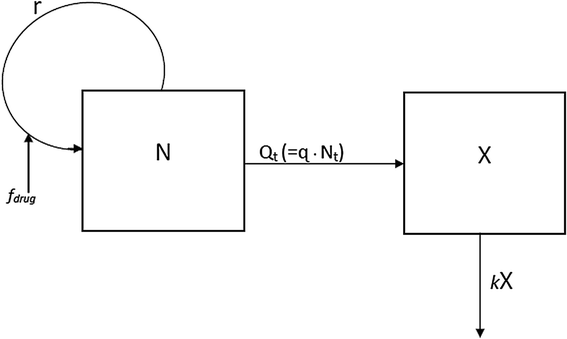

Supplement: Supplementary file 2 — Authors’ original file for figure 1 [file 40203_2014_3_MOESM2_ESM.gif]

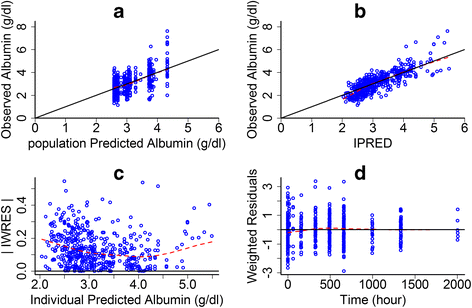

Supplement: Supplementary file 3 — Authors’ original file for figure 2 [file 40203_2014_3_MOESM3_ESM.gif]

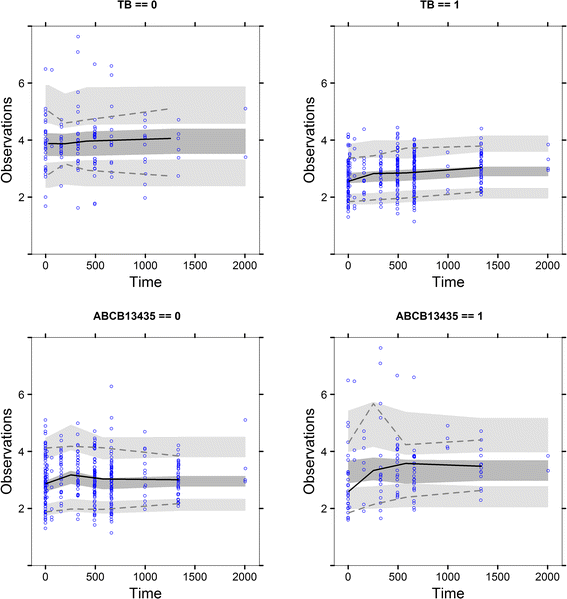

Supplement: Supplementary file 4 — Authors’ original file for figure 3 [file 40203_2014_3_MOESM4_ESM.gif]

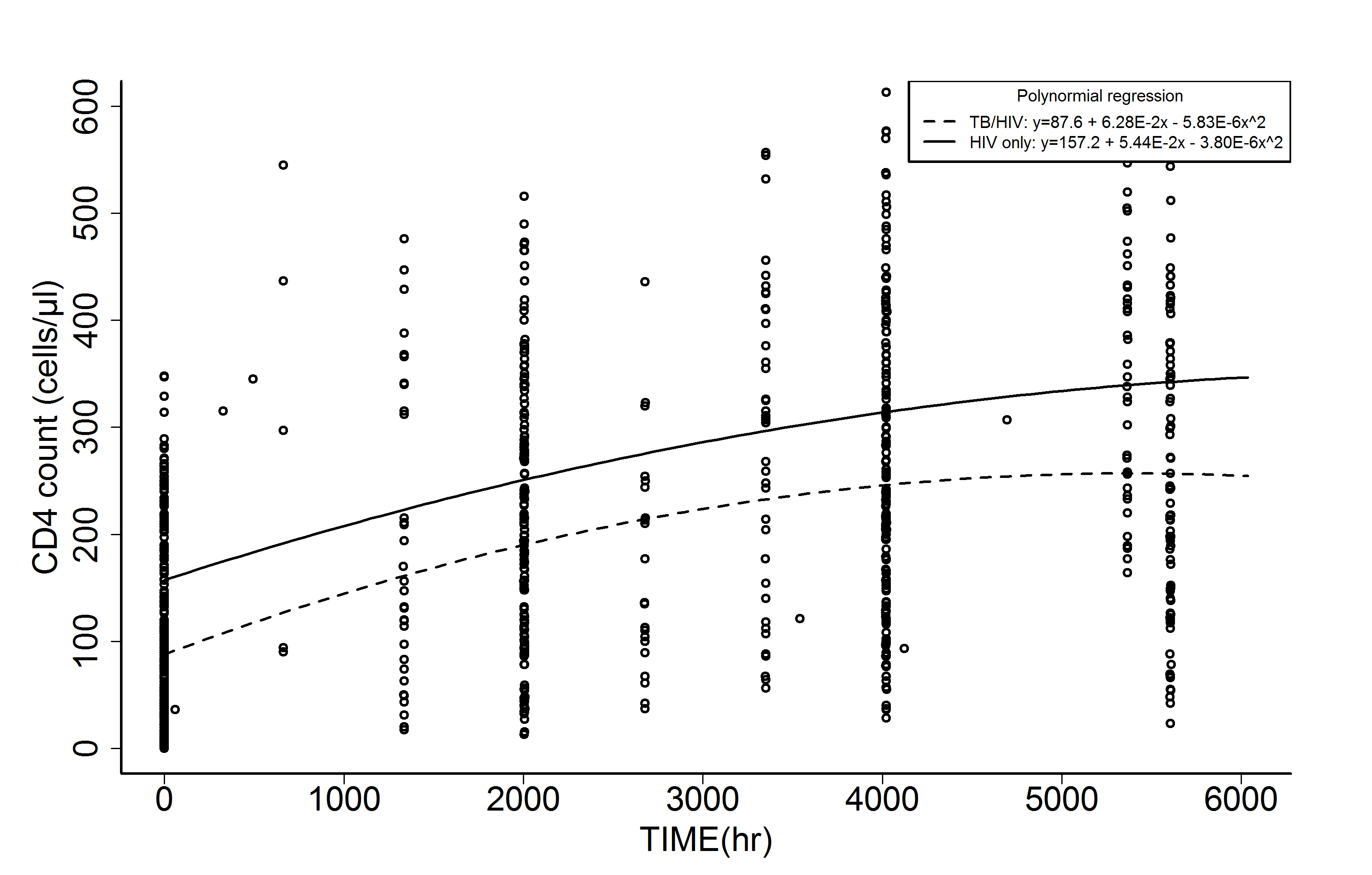

Supplement: Supplementary file 5 — Authors’ original file for figure 4 [file 40203_2014_3_MOESM5_ESM.tiff]
